# Specific Deubiquitinating Enzymes Promote Host Restriction Factors Against HIV/SIV Viruses

**DOI:** 10.3389/fimmu.2021.740713

**Published:** 2021-09-22

**Authors:** Wenying Gao, Yajuan Rui, Guangquan Li, Chenyang Zhai, Jiaming Su, Han Liu, Wenwen Zheng, Baisong Zheng, Wenyan Zhang, Yongjun Yang, Shucheng Hua, Xiaofang Yu

**Affiliations:** ^1^Center for Pathogen Biology and Infectious Diseases, Institute of Virology and AIDS Research, The First Hospital of Jilin University, Changchun, China; ^2^Cancer Institute (Key Laboratory of Cancer Prevention and Intervention, Ministry of Education), Second Affiliated Hospital, School of Medicine, Zhejiang University, Hangzhou, China; ^3^Jilin Provincial Key Laboratory on Molecular and Chemical Genetics, The Second Hospital of Jilin University, Changchun, China; ^4^Department of Respiratory Medicine, The First Hospital of Jilin University, Changchun, China; ^5^Key Laboratory of Zoonosis, Ministry of Education, College of Veterinary Medicine, Jilin University, Changchun, China

**Keywords:** USP8, deubiquitinating enzymes, antiviral activity, viral proteins, ubiquitin ligase

## Abstract

Hijacking host ubiquitin pathways is essential for the replication of diverse viruses. However, the role of deubiquitinating enzymes (DUBs) in the interplay between viruses and the host is poorly characterized. Here, we demonstrate that specific DUBs are potent inhibitors of viral proteins from HIVs/simian immunodeficiency viruses (SIVs) that are involved in viral evasion of host restriction factors and viral replication. In particular, we discovered that T cell-functioning ubiquitin-specific protease 8 (USP8) is a potent and specific inhibitor of HIV-1 virion infectivity factor (Vif)-mediated apolipoprotein B mRNA-editing enzyme catalytic polypeptide-like 3 (APOBEC3)G (A3G) degradation. Ectopic expression of USP8 inhibited Vif-induced A3G degradation and suppressed wild-type HIV-1 infectivity even in the presence of Vif. In addition, specific DUBs repressed Vpr-, Vpu-, and Vpx-triggered host restriction factor degradation. Our study has revealed a previously unrecognized interplay between the host’s DUBs and viral replication. Enhancing the antiviral activity of DUBs therefore represents an attractive strategy against HIVs/SIVs.

## Introduction

Ubiquitin modification of proteins regulates their functions and is involved in virtually all aspects of cellular processes (1). Ubiquitin modification is further regulated by deubiquitinating enzymes (DUBs). DUBs have been divided according to active site homology into six broad classes (2): Ub-specific proteases (USPs), Ub C-terminal hydrolases (UCHs), ovarian tumor proteases (OTUs), Machado–Joseph disease protein domain proteases, JAMM/MPN domain-associated metallopeptidases (JAMMs), and monocyte chemotactic protein-induced proteins (MCPIPs) (3). Within the six classes of DUBs, USPs are highly diversified, consisting of more than 50 members. Many studies have reported mutations in USPs involved in multiple biological processes (4). However, the role of DUBs in the interplay between viruses and the host is poorly characterized.

Ubiquitin modification of proteins regulates their function (1, 4, 5) and the reverse process, deubiquitination, is also important for many biological events (6). The importance of deubiquitylating enzyme function is underscored by its frequent deregulation in human diseases such as cancer, infections, and neurological disease, making these enzymes potential drug targets. Also, hijacking host ubiquitin pathways is essential for the replication of diverse viruses (7, 8). The host’s cytidine deaminase apolipoprotein B mRNA-editing enzyme catalytic polypeptide-like 3 (APOBEC3) proteins are potent inhibitors of virion infectivity factor (Vif)-deficient human immunodeficiency virus 1 (HIV-1ΔVif) (9, 10). APOBEC3B/DE/G/F (A3B/DE/G/F) proteins become packaged into HIV-1 virions during virus production and inhibit viral reverse transcription in newly infected target cells. Other APOBEC3 protein family members, including APOBEC3A (A3A), APOBEC3C (A3C), and APOBEC3H (A3H), also impair HIV-1 replication by packaging into virions (11). The Vif protein of HIV-1 neutralizes APOBEC3’s antiviral functions by forming a viral-specific CRL5 E3 ubiquitin ligase complex consisting of CUL5, ElOB/C, and CBFβ (12–14) to promote the polyubiquitination and degradation of APOBEC3 substrates (15).

Three other HIV/simian immunodeficiency virus (SIV) accessory proteins, Vpu, Vpr, and Vpx, are also essential components of viral replication and pathogenesis. Ubiquitin modification is vital for them on the process of target protein destruction. HIV-1 Vpu hijacks CUL1 and β-TrCP to form a CRL1 E3 ubiquitin ligase complex, triggering the degradation of BST-2 (16) and PSGL-1 (17). Vpr recruits DCAF1, DDB1, and CUL4 to form the CRL4 E3 ubiquitin ligase complex to induce the degradation of HLTF (18). Vpx proteins of HIV-2Rod and certain SIVs overcome the antiviral function of SAMHD1 in myeloid cells by inducing its polyubiquitination and degradation (19, 20).

In this report, we demonstrate that DUBs are potent inhibitors of viral evasion of host restriction. We have identified various DUBs inhibiting functions of HIV Vif, Vpr, Vpu, and Vpx proteins. Collectively, our study reveals a previously unrecognized interplay between the host’s DUBs and viral replication. Enhancing the antiviral activity of DUBs therefore represents an attractive strategy against HIVs/SIVs.

## Results

### T Cell-Specific USP8 Inhibits Vif-Induced A3G Degradation and Suppresses Wild-Type HIV-1 Infectivity

**Figure 1:**
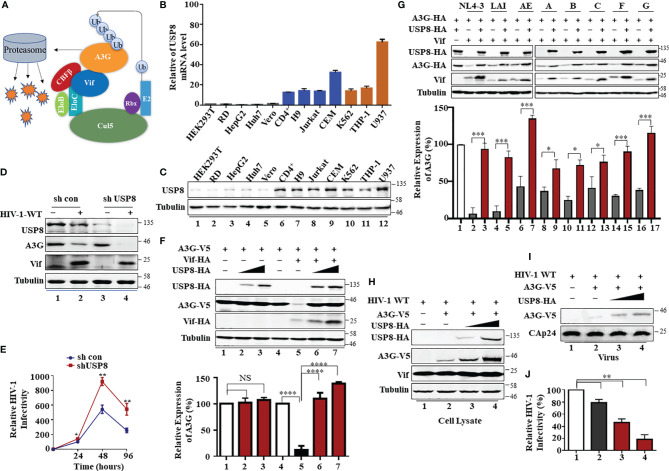
T cell-specific ubiquitin-specific protease 8 (USP8) inhibits virion infectivity factor (Vif)-induced apolipoprotein B mRNA-editing enzyme catalytic polypeptide-like 3 (APOBEC3)G (A3G) degradation and suppresses wild-type (WT) HIV-1 infectivity. **(A)** A model of HIV Vif assembly with the Cul5, CBFβ, and EloB/C E3 components to mediate polyubiquitination and degradation of the A3G protein. **(B)** USP8 mRNA expression levels in various cell types were detected by RT-qPCR. USP8 mRNA expression in HEK293T cell line was set to 1.0. Glyceraldehyde 3-phosphate dehydrogenase (GAPDH) was used as a loading control. Orange, myeloid cell lines; blue, CD4^+^ T cell lines; black, other cell lines. **(C)** USP8 protein expression levels in various cell types were detected by immunoblotting. **(D)** The effect of USP8 silencing on A3G expression in HIV-1-infected H9 cell. H9 USP8 silencing cells were infected with HIV or not for 48 h. Endogenous USP8 and A3G were analyzed by immunoblotting. Virus infection was determined by Pr55Gag. **(E)** The effect of USP8 silencing on HIV infectivity in H9 cell. USP8 silencing and its control H9 cells were infected with HIV for 30 h. The cells were then washed three times with phosphate buffered saline (PBS) and placed in fresh RPMI-1640 medium with 10% fetal bovine serum (FBS). Cell supernatants were then harvested after 24, 48, and 96 h of infection. Virus infectivity was assessed using TZM-BL indicator cells. **(F)** USP8 inhibits Vif-mediated A3G degradation. HEK293T cells were transfected with A3G-HA and Vif-HA or its empty vector in the presence of increasing amounts of USP8-HA. Cells were harvested 48 h after transfection. Protein expression in the cell lysates was analyzed by immunoblotting with anti-HA antibody targeting USP8-HA, A3G-HA, and Vif-HA proteins. Tubulin was used as a loading control. Quantification of A3G expression was analyzed by ImageJ2X. A3G expression alone was normalized to 100%. **(G)** USP8 repressed the anti-A3G activity of Vif molecules from diverse HIV-1 subtypes. HEK293T cells were transfected with A3G-HA and diverse HIV-1 Vif subtypes or empty vector in the presence or absence of USP8. Cells were harvested 48 h after transfection, and protein expression in the cell lysates was analyzed by immunoblotting with anti-HA antibody targeting USP8-HA, A3G-HA, and anti-Vif antibody targeting Vif proteins. Tubulin was used as a loading control. Quantification of A3G expression was analyzed by ImageJ2X. A3G expression alone was normalized to 100%. **(H–J)** HIV-1 infectivity was significantly reduced when USP8 was co-expressed with A3G. WT HIV-1 and A3G-HA or control vector were co-transfected into HEK293T cells with increasing amounts of USP8-HA. After 48 h, cells were harvested, and protein expression was analyzed by immunoblotting with anti-Pr55Gag and anti-HA antibody targeting USP8-HA and A3G-HA proteins. Tubulin was used as a loading control **(H)**. Virion particle-containing supernatants were harvested and filtered through a 0.45-μm filter, then concentrated by ultracentrifugation. Virion pellets were immunoblotted with anti-CAp24, anti-Vif, and anti-HA antibody targeting A3G-HA **(I)**. HIV-1 infectivity was assessed by TZM-bl indicator cells **(J)**. WT HIV-1 infectivity alone was set to 100% **(J)**. Column results were from n = 3 independent biological experiments **(A, F, G, J)**, and immunoblotting results are representative of n = 3 experiments **(F, H)**. Means and standard deviations are presented. The statistical significance analyses were performed using two-sided unpaired t-tests (NS, not significant, *p < 0.05, **p < 0.01, ***p < 0.001, ****p < 0.0001).

The deubiquitination enzyme USP8 is a human T cell-specific factor regulating T-cell maturation and functions (21, 22), and CD4^+^ T cells are primary targets of HIV-1 infection. HIV-1 is potently inhibited by several human cytidine deaminases that are members of human APOBEC3 proteins. HIV-1 Vif neutralizes some APOBEC3’s antiviral functions by forming viral-specific CRL5 E3 ubiquitin ligase complexes (12, 13, 23, 24) to promote the polyubiquitination and degradation of APOBEC3 substrates (**Figure 1A**). Whether any host factors involved in the deubiquitination pathway can influence Vif function has not been explored.

We observed that USP8 is highly expressed in primary CD4^+^ T cells and myeloid cells compared with HEK293T cells and RD cells (**Figures 1B, C**). Primary CD4^+^ T cells and myeloid cells are the preferred target cells of HIV-1. We screened a library of mammalian expression vectors that encode 32 USPs twice and discovered USP8 is distinguished from the group. USP8 significantly impaired Vif-triggered APOBEC3G (A3G) degradation (**Supplementary Figures S1H, I**). To investigate whether endogenous USP8 in T cells has an effect on Vif-induced A3G degradation, we generated USP8 knocking down stable cell line in H9 T cells (**Supplementary Figure S1A**). shCon and shUSP8 H9 cells were infected with wild-type (WT) HIV-1 at the same titer or mock infected. As shown in **Figure 1D**, silencing USP8 has no effect on endogenous A3G expression (lanes 1 and 3). A3G was degraded during HIV-1 infection (lanes 1 and 2). Surprisingly, A3G degradation was enhanced in virus-infected shUSP8 H9 cells (lanes 2 and 4). In addition, cell supernatants were collected at different time points, and virus infectivity was assessed using TZM-BL indicator cells. We found that USP8 knocking down had a positive effect on HIV-1 progeny virus replication (**Figure 1E**). A similar result was also determined in primary CD4^+^ cells (**Supplementary Figure S1B**). Since USP8 expression in HEK293T cells is lower than that in CD4^+^ T cells, we detected whether increasing the USP8 expression could affect HIV-1 Vif function in HEK293T cells. As a result, increasing the USP8 expression effectively inhibited HIV-1 Vif-induced A3G degradation (**Figure 1F**). In the absence of exogenous USP8 expression, HIV-1 Vif induced a >70% reduction in A3G (**Figure 1F**, lane 5) when compared to control cells (**Figure 1F**, lane 4). The Vif-induced degradation of A3G was essentially abolished in the presence of increasing amounts of USP8 (**Figure 1F**, lanes 6 and 7). In the absence of Vif, A3G expression was not affected by USP8 (**Figure 1F**, lanes 1–3). Consistent with previous reports, we also observed that USP8 could rescue Vif expression in a dose-dependent manner, since Vif was ubiquitinated and degraded *via* the proteasome pathway (25) (**Figure 1F**). Furthermore, silencing endogenous USP8 expression (**Supplementary Figure S1C**) enhanced HIV-1 Vif-mediated A3G degradation in HEK293T cells (**Supplementary Figures S1C, D**). The ability of HIV-1 Vif to counteract A3G is quite conserved among different HIV-1 subtypes. To investigate whether USP8 blocking of anti-A3G activity of Vif is universal, seven viral isolates from patients representing seven HIV-1 subtypes Vif molecules were selected (26, 27), except NL4-3 Vif. We validated that USP8 has the ability to block the anti-A3G activity of Vif molecules from diverse HIV-1 subtypes (**Figure 1G**).

To examine the possible effect of USP8 on HIV-1 infection, we first co-expressed increasing amounts of USP8 with HIV-1 (NL4-3). USP8 expression had no detectable effect on viral protein expression, as determined by the expression of intracellular HIV-1 Pr55Gag in HEK293T cells (**Supplementary Figure S1E**). Virus release (**Supplementary Figure S1F**) and the infectivity of the released virus (**Supplementary Figure S1G**) were also unaffected by USP8 expression in the absence of A3G. In contrast, in the presence of USP8, intracellular A3G expression (**Figure 1H**) and virion incorporation of A3G (**Figure 1I**) were increased, even in the presence of HIV-1 Vif. At the same time, the infectivity of the released HIV-1 was reduced in the presence of USP8 (**Figure 1J**), which is consistent with the increased amount of A3G virion packaging. Combined with these data, these results indicate that USP8 exerts its potent antiviral effect through Vif. USP8 antagonized the ability of HIV-1 Vif to suppress the antiviral function of A3G, resulting in an enhanced antiviral activity of A3G against Vif-containing HIV-1.

### The Active Site of the USP8 Enzyme Is Required for Inhibition of HIV-1 Vif Function

**Figure 2:**
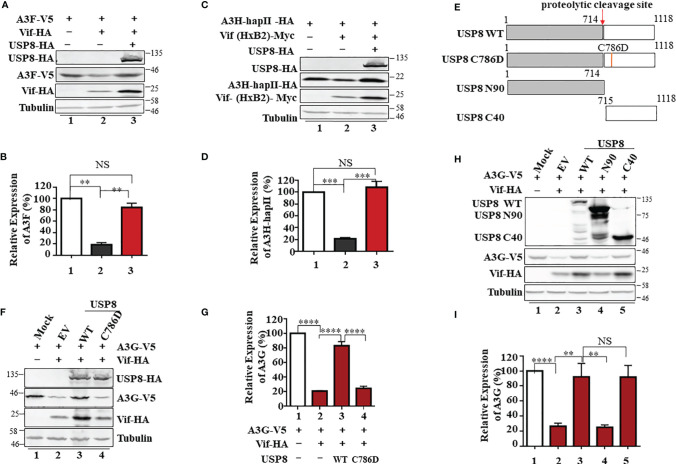
The active site of ubiquitin-specific protease 8 (USP8) is required for inhibition of HIV-1 virion infectivity factor (Vif) function. USP8 efficiently inhibited HIV-1 Vif-induced apolipoprotein B mRNA-editing enzyme catalytic polypeptide-like 3 (APOBEC3)F (A3F) **(A)** and A3H-HapII **(C)** degradation. **(A, C)** HEK293T cells were co-transfected with expression vector as indicated. Cells were harvested 48 h after transfection. Protein expression in the cell lysates was analyzed by immunoblotting with the corresponding antibodies. Tubulin was used as a loading control. **(B, D)** Quantification of A3F or A3H-HapII expression was analyzed by ImageJ2X. A3F **(B)** or A3H-HapII **(D)** expression alone was normalized to 100%. **(E)** The schematic represents USP8 wild type (WT) and mutations used in the study. The red arrow shows the USP8 proteolytic cleavage site (p.714Arg). **(F)** The USP8C786D mutation has lost the ability to inhibit Vif-mediated degradation of A3G. HEK293T cells were transfected with A3G-V5 alone or together with Vif-HA in the presence of USP8 WT-HA, the USP8C786D-HA mutation, or empty vector (EV). Cells were harvested 48 h after transfection, and protein expression in the cell lysates was analyzed by immunoblotting with anti-V5 and anti-HA antibodies targeting A3G-V5, USP8-HA, and Vif-HA protein. Tubulin was used as a loading control. **(G)** Quantification of A3G expression was analyzed by ImageJ2X. A3G expression alone was normalized to 100%. **(H)** HEK293T cells were transfected with A3G-V5 alone or together with Vif-HA in the presence of USP8 WT-HA, a USP8 truncation, or EV. Cells were harvested after 48 h, and protein expression in the cell lysates was analyzed by immunoblotting with the corresponding antibodies. **(I)** Quantification of A3G expression was analyzed by ImageJ2X. A3G expression alone was normalized to 100%. Column results were from n = 3 **(B, D, G, I)** independent experiments, and immunoblotting results are representative of n = 3 experiments **(A, C, F, H)**. Means and standard deviations are presented. The statistical significance analyses were performed using two-sided unpaired t-tests (NS, not significant; **p < 0.01, ***p < 0.001, ****p < 0.0001).

In addition to A3G, A3H-Haplotype II (hap II), which is overcome by HxB2 Vif (26), and A3F are also potent inhibitors of HIV-1 that are neutralized by Vif (13, 28, 29). We observed that USP8 can efficiently inhibit HIV-1 Vif-induced degradation of A3F (**Figures 2A, B**) and A3H-HapII (**Figures 2C, D**). The degradation of other known HIV-1 Vif targets, such as A3C (**Supplementary Figures S2A, B**) and A3DE (**Supplementary Figures S2C, D**), was also inhibited by USP8. Non-primate lentiviral Vif from bovine immunodeficiency virus (BIV) targets cow APOBEC3 proteins (30). Unlike HIV-1 Vif, non-primate lentivirus BIV Vif assembles with CUL2 and the ElOB/C E3 component (**Supplementary Figure S2E**) and is not regulated by CBFβ to trigger the degradation of bovine APOBEC3 (31). Since USP8 sequence is highly conserved between cow and human (**Supplementary Figure S2F**), human USP8 was also able to block BIV Vif-induced degradation of cow APOBEC3 in HEK293T cells (**Supplementary Figures S2G, H**). Importantly, we observed that diverse Vif expression could be enhanced by USP8 (**Supplementary Figures S2A, C, G**). Taken together, USP8 targets diverse Vif viral substrate receptors but not the E3 cellular components.

To identify whether the deubiquitinating enzymatic activity of USP8 is required for its antagonism of Vif function, a key residue Cysteine 786 of USP8 is mutated to abolish its enzymatic activity (21) (**Figure 2E**). We found that the active site mutant USP8 C786D lost the ability to inhibit Vif-mediated A3G degradation (**Figure 2F**, lane 4) when compared to wild-type USP8 (**Figure 2F**, lane 3). In the presence of USP8 C786D, Vif-mediated A3G degradation was as efficient as the no-USP8 control (**Figure 2G**, bar 4 vs. bar 2). USP8 is vital for the development and homeostasis of T cells and is cleaved in activated CD4^+^ T cells by caspases. The C-terminal fragment of USP8, but not N-terminal fragment, possesses the enzymatic activity (21). Somatic mutations in USP8 enhance its proteolytic cleavage and are associated with Cushing’s disease resulting from elevated deubiquitinase activity (22). We observed that the C-terminal fragment (C40) but not the N-terminal fragment (N90) of USP8 maintained the inhibitory activity against HIV-1 Vif (**Figures 2H, I**). Collectively, enzymatic activity of USP8 is critical for the inhibitory effect of Vif function.

### USP8 Specifically Interacts With HIV-1 Vif and Reduces Vif-Triggered A3G Polyubiquitination

**Figure 3:**
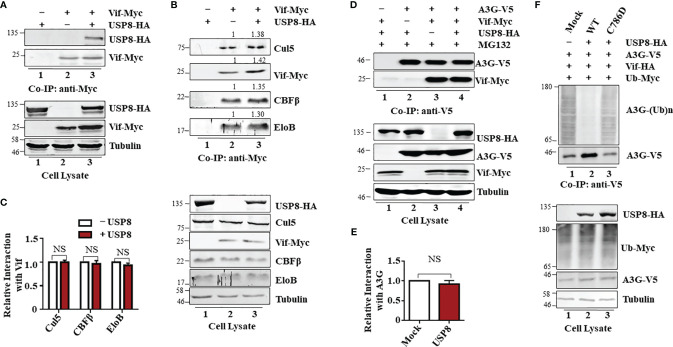
Ubiquitin-specific protease 8 (USP8) specifically interacts with HIV-1 virion infectivity factor (Vif) and reduces Vif-triggered apolipoprotein B mRNA-editing enzyme catalytic polypeptide-like 3 (APOBEC3)G (A3G) polyubiquitination. **(A)** Co-precipitation of USP8 with Vif. HEK293T cells were transfected with USP8-HA, Vif-Myc alone, or both, as indicated. Cell lysates were prepared and immunoprecipitated using anti-Myc antibody conjugated to agarose beads 48 h after transfection. Cell lysates and precipitated samples were separated by sodium dodecyl sulfate–polyacrylamide gel electrophoresis (SDS-PAGE), transferred to nitrocellulose membranes, and reacted with an anti-HA antibody to detect USP8-HA and an anti-Myc antibody to detect Vif-Myc. Tubulin was used as the loading control for the cell lysate. **(B)** USP8 does not affect Vif–CRL5 E3 ubiquitin ligase formation. HEK293T cells were transfected with Vif-Myc, USP8-HA, or both. Cell lysates were immunoprecipitated with anti-Myc antibodies conjugated to agarose beads. Cell lysates and precipitated samples were analyzed by immunoblotting with the corresponding antibodies. Tubulin was used as the loading control for the cell lysate. **(C)** Relative binding ability of Vif with Cul5, CBFβ, and EloB in the presence or absence of USP8 was determined by ImageJ2X. Protein binding to Vif in lane 2 **(B**, upper blots**)** was set to 1.0. Data are means ± SD from n = 3 independent experiments. The statistical significance analyses were performed using two-sided unpaired t-tests (NS, not significant). **(D)** The association of Vif with A3G is also not affected by USP8. HEK293T cells were co-transfected with expression vectors as indicated. Cells were treated with 10 mM MG132 12 h before harvesting. Vif protein was immunoprecipitated from cell lysates with an anti-V5 antibody conjugated to agarose beads. Cell lysates and precipitated samples were analyzed by immunoblotting with the corresponding antibodies. Tubulin was used as the loading control for the cell lysate. **(E)** Quantification of co-precipitated Vif relative to A3G was determined by ImageJ2X. Data are means ± SD from n = 3 independent experiments. The statistical significance analyses were performed using two-sided unpaired t-tests (NS, not significant). **(F)** USP8 inhibits Vif-triggered polyubiquitination of A3G. HEK293T cells were transfected with the empty vector, Vif-HA, A3G-V5, Ub-Myc, or USP8-HA as indicated. Cells were treated with 10 mM MG132 for 12 h before harvesting. Cell lysates were prepared and immunoprecipitated using anti-V5 antibody conjugated to agarose beads 48 h after transfection. Cell lysates and precipitated samples were analyzed by immunoblotting with the corresponding antibodies. Immunoblotting results from panels **(A, B, D, F)** are representative of n = 3 independent experiments.

To explore the mechanism of USP8-mediated Vif inhibition, we employed co-immunoprecipitation (co-IP) assay to determine whether USP8 could interact with HIV-1 Vif. In the absence of Vif, USP8 was not detected in the co-IP sample (**Figure 3A**, lane 1), indicating the specificity of the assay system. In the presence of Vif, co-precipitation of USP8 with Vif was detected (**Figure 3A**, lane 3). Gads is a binding partner of USP8, which is required for USP8 in regulating the development and homeostasis of T cells (21). The interaction with HIV-1 Vif reduced the ability of USP8 binding with its functional cofactor Gads (**Supplementary Figures S3A, B**). These data establish a specific interaction between HIV-1 Vif and USP8.

Vif forms a viral-specific E3 ubiquitin ligase complex with cellular proteins CUL5, ElOB/C, and CBFβ to trigger polyubiquitination of substrates (12). Vif interacts with USP8; we next assessed whether USP8 disrupts Vif–Cullin 5–E3 ubiquitin ligase complex assembly. Interestingly, USP8 did not interact with CUL5, ElOB, or CBFβ (**Supplementary Figure S3C**) in the absence of Vif. The ability of Vif to interact with CUL5, ElOB, and CBFβ (**Figure 3B**, lane 2; **Figure 3C**) was not affected by USP8 overexpression (**Figure 3B**, lane 3; **Figure 3C**). The interaction of Vif with the target protein A3G (**Figure 3D**, lane 3; **Figure 3E**) was also not affected by USP8 (**Figure 3D**, lane 4; **Figure 3E**). However, HIV-1 Vif-induced polyubiquitination of A3G (**Figure 3F**, lane 1) was significantly reduced in the presence of USP8 (**Figure 3F**, lane 2). In contrast, enzymatically defective USP8 C786D, which could not inhibit Vif-triggered A3G degradation, was disabled in suppressing HIV-1 Vif-induced polyubiquitination of A3G (**Figure 3F**, lane 3) when compared to the WT USP8 (**Figure 3F**, lane 2). Additionally, we confirmed that USP8 repressed Vif-mediated A3G polyubiquitination *in vitro* study (**Supplementary Figures S3D–F**). Ubiquitinated A3G was purified from HEK293T cells transfected with Ub-Flag, Vif-HA, and A3G-V5 using anti-V5 affinity purification. WT USP8 and functional dominant truncation C40, which purified from HEK293T cells and incubated with ubiquitinated A3G, decreased A3G polyubiquitination, while enzymatic mutation C786D was disabled (**Supplementary Figure S3D**). USP8 C40 purified from *Escherichia coli* reduced A3G polyubiquitination in a time-dependent fashion (**Supplementary Figure S3E**). Likewise, USP8 C40^C786D^, an enzymatic mutant, lost the ability of A3G deubiquitination (**Supplementary Figure S3F**). Together, these results indicated that USP8 specifically interacts with Vif and deubiquitinates the ubiquitination of A3G mediated by Vif.

### HIV-1 Antagonizes USP8-Mediated Viral Suppression by Impairing USP8 Expression in CD4^+^ Cells

**Figure 4:**
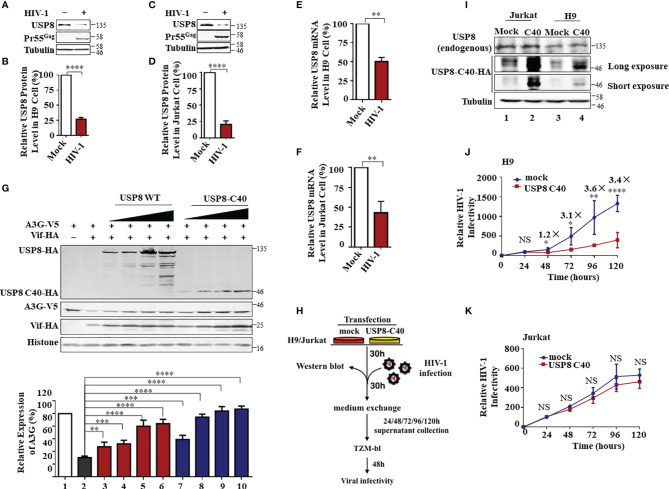
HIV-1 antagonizes ubiquitin-specific protease 8 (USP8) suppression by lowering USP8 protein levels in CD4^+^ cells. **(A–D)** HIV-1 suppresses USP8 expression at the protein level. H9 **(A)** and Jurkat **(C)** cells were infected or not infected with wild-type (WT) HIV for 48 h. Endogenous USP8 was analyzed by immunoblotting. Virus infection was determined by the presence of Pr55Gag. Tubulin was used as a loading control. USP8 expression was measured by ImageJ2X (mock infection was set to 100%) **(B, D)**. **(E, F)** HIV-1 suppresses USP8 expression at the mRNA level. H9 **(E)** and Jurkat **(F)** cells were infected or not infected with WT HIV for 48 h. USP8 mRNA expression was detected by RT-qPCR. Glyceraldehyde 3-phosphate dehydrogenase (GAPDH) was used as a control. **(G)** Comparison of the effect of full-length USP8 and C40-truncated USP8 on virion infectivity factor (Vif)-mediated apolipoprotein B mRNA-editing enzyme catalytic polypeptide-like 3 (APOBEC3)G (A3G) degradation. A3G-V5, together with Vif-HA or its control vector, was transfected into HEK293T cells in the presence of increasing amounts of full-length USP8 or C40-truncated USP8. Cells were harvested after 48 h, and cell lysates were heated with lysis buffer, then immunoprecipitated with the corresponding antibodies. Quantification of A3G expression was analyzed by ImageJ2X. A3G expression alone was normalized to 100%. **(H)** Workflow for USP8 C40 inhibition of HIV infectivity in H9 and Jurkat cells. **(I)** USP8-C40-HA or control vector was electro-transfected into H9 **(I)** or Jurkat **(J)** cells. After 30 h, USP8 and USP8 C40-HA expression was analyzed by immunoblotting. **(J, K)** USP8 C40-HA or control vector was electro-transfected into H9 **(K)** or Jurkat **(L)** cells. After 30 h, H9/Jurkat cells were infected with WT HIV for another 30 h. The cells were then washed three times with phosphate buffered saline (PBS) and placed in fresh RPMI-1640 medium with 10% fetal bovine serum (FBS). Cell supernatants were then harvested after 24, 48, 72, 96, and 120 h of infection. Virus infectivity was assessed using TZM-BL indicator cells. Means and standard deviations are presented. Panels **(B, D–F, J, K)** are results from n = 3 independent experiments. The statistical significance analyses were performed using two-sided unpaired t-tests (NS, not significant; *p < 0.05; **p < 0.01; ***p < 0.001; ****p < 0.0001).

Although USP8 exhibits strong inhibitory activity against Vif-mediated A3G degradation (**Figure 1F**), the anti-HIV-1 function of USP8 could be compromised during HIV-1 replication. Because USP8 expression was reduced in HIV-1-infected CD4^+^ T cells, which is based on mass spectrometry data published by several groups (17, 32, 33). In both H9 (**Figures 4A, B** and **Supplementary Figure S4A**) and Jurkat (**Figures 4C, D** and **Supplementary Figure S4B**) CD4^+^ T cells, HIV-1 infection resulted in depletion of USP8 proteins significantly. Moreover, the USP8 mRNA level was also reduced in HIV-1-infected T cells (**Figures 4E, F**), suggesting that down-modulation of USP8 expression by HIV-1 is at least partially the result of an alteration in USP8 mRNA transcription or RNA stability. The C-terminal fragment of USP8 contains the deubiquitining enzymatic activity (22) and is a potent inhibitor of HIV-1 Vif function (**Figure 2H**). HIV-1 Vif-triggered A3G degradation was blocked slightly more efficiently by USP8 C40 than by full-length USP8 (**Figure 4G**), which was correlated with the inhibition of HIV-1 Vif-induced A3G polyubiquitination (**Supplementary Figures S3D**, **S4C, D**). To further validate USP8 C40 function during HIV-1 infection, USP8 C40 was transduced into CD4^+^ T cells (**Figure 4H**). Transduction with USP8 C40 in H9 cells that express endogenous APOBEC3 antiviral proteins (**Figure 4I**, lanes 3 and 4) significantly inhibited HIV-1 replication (**Figure 4J**). In contrast, USP8 C40 (**Figure 4I**, lanes 1 and 2) has no effect on HIV-1 replication (**Figure 4K**) when transduced in APOBEC3-negative Jurkat cells. These data demonstrate that modulation of USP8 function in CD4^+^ T cells can enhance the antiviral activity of APOBEC3 cytidine deaminases against HIV-1 even in the presence of Vif.

### Specific Deubiquitinating Enzymes Suppress Different HIV/SIV Accessory Protein-Mediated Degradation of Host Restriction Factors

**Figure 5:**
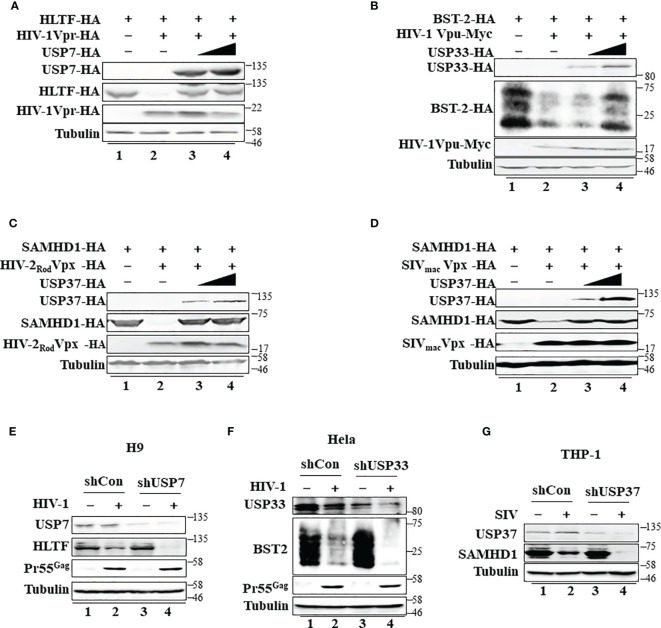
Specific deubiquitinating enzymes suppress degradation of host restriction factors mediated by HIV/simian immunodeficiency virus (SIV) proteins. **(A)** Ubiquitin-specific protease 7 (USP7) inhibits HIV-1 Vpr-induced degradation of HLTF. HEK293T cells were transfected with expression vectors as indicated. Cell lysates were immunoblotted with the corresponding antibodies. **(B)** USP33 inhibits HIV-1 Vpu-induced BST-2 degradation. The experimental methods were the same as in panel **(A). (C, D)** USP37 inhibits HIV-2 Rod Vpx- **(C)** or SIV mac Vpx **(D)**-induced SAMHD1 degradation. The experimental methods were the same as in panel **(A). (E)** The effect of USP7 silencing on HLTF expression in WT HIV-1-infected H9 cell. H9 shUSP7 and its control cell lines were infected with WT HIV-1 or not for 48 h. Endogenous USP7 and HLTF were analyzed by immunoblotting. Virus infection was determined by Pr55Gag. **(F)** The effect of USP33 silencing on BST2 expression in WT HIV-infected Hela cell. The process is described in panel **(E). (G)** The effect of USP33 silencing on SAMHD1 expression in SIVmac-GFP-infected THP-1 cell. THP-1 cells stably expressed USP33shRNA. USP33 knockdown THP-1 cells were differentiated into macrophages by phorbol 12-myristate 13-acetate (PMA) treatment and infected with SIV mac viruses for 24 h. Endogenous USP33 and SAMHD1 were analyzed by immunoblotting. Immunoblotting results are representative of n = 3 independent experiments.

Ubiquitin modification is linked to many cellular processes. Diverse DUBs are involved in reversing protein ubiquitination and therefore modulate the outcome of this posttranslational modification. HIV-1 Vpr, HIV-1 Vpu, and HIV-2 Rod/SIVmac Vpx also form virus-specific E3 ubiquitin ligase complexes to mediate the polyubiquitination and degradation of various target proteins (**Supplementary Figures S5A–C**). However, in contrast to USP8-mediated Vif inhibition, USP8 had a little effect on HIV-1 Vpr-mediated HLTF degradation (**Supplementary Figure S5G**), HIV-1 Vpu-mediated BST-2 depletion (**Supplementary Figure S5H**), or HIV-2Rod/SIVmac Vpx-mediated SAMHD1 degradation (**Supplementary Figures S5I, J**). Although USP8 had little effect on HIV-1 Vpr, HIV-1 Vpu, or HIV-2 Rod/SIVmac Vpx function, we identified other potent DUB inhibitors of HIV-1 Vpr, HIV-1 Vpu, and HIV-2 Rod/SIVmac Vpx (**Figures 5A–D**) after screening 32 USPs (**Supplementary Figures S5D–F**). USP7 inhibited HIV-1 Vpr-mediated HLTF degradation (**Figure 5A**), USP33 inhibited HIV-1 Vpu-mediated BST-2 depletion (**Figure 5B**), and USP37 inhibited HIV-2 Rod/SIVmac Vpx-mediated SAMHD1 degradation (**Figures 5C, D**). Moreover, we investigated different USP functions in suitable cell lines as previously reported (16, 20, 34). Silencing USP7 in H9 T cells, HLTF degradation was more efficient in shUSP7 cells in the presence of HIV-1 infection compared with its control cells (**Figure 5E**, lanes 2 and 4). As a result, silencing USP7 promoted HIV-1 infectivity (**Supplementary Figure S5K**). Meanwhile, similar result was demonstrated in shUSP33 HeLa cells that BST2 degradation was enhanced during HIV-1 infection (**Figure 5F**, lanes 2 and 4) and subsequently promoted HIV-1 infectivity (**Supplementary Figure S5L**). HIV-1 Pr55Gag was determined to demonstrate silencing USPs has no effect on virus replication (**Figures 5E, F**). SAMHD1 was degraded in THP-1 cells when infected with SIVmac virus. As expected, SIV-induced SAMHD1 degradation was more efficient in the absence of USP37 THP-1 cells (**Figure 5G**). Collectively, specific DUBs play important roles in virus–host relationships.

## Discussion

APOBEC3 cytidine deaminases are host restriction factors against HIV-1 and related retroviruses. HIV-1 Vif targets APOBEC3 for polyubiquitination and subsequent degradation to ensure successful viral replication. A3G protein degradation by ubiquitination was mediated by Vif- and cullin-RING-independent pathway. It is reported that USP49 is a new antiviral factor. USP49 increased A3G protein expression by removing ubiquitin and enhanced its anti-HIV-1 activity (35). Different from USP49, we discovered that USP8 is a potent and specific inhibitor of Vif. USP8 overexpression alone or knocking down has no effect on A3G protein stability (**Figures 1D, F**). Notably, USP8 bound Vif (**Figure 3A**) without disturbing Vif–Cullin-RING E3 complex assembly (**Figure 3B**) or Vif–A3G interaction (**Figure 3D**). USP8 blocked Vif-induced polyubiquitination and degradation of A3G (**Figures 1D–H**, **3F**). Consequently, USP8 could attenuate the infectivity of HIV-1 in the presence of A3G and suppress HIV-1 replication in CD4^+^ T cells (**Figure 4J**). Meanwhile, both mRNA and protein expression levels of USP8 were significantly downregulated during HIV-1 infection in T cells (**Figures 4A–D** and **Supplementary Figures S4A, B**), which indicated that HIV-1 has evolved new antagonisms against USP8.

We also observed that diverse Vif expression could be enhanced by USP8 (**Figures 2A, C, F**, **3H** and **Supplementary Figures S2A, C, G**). Importantly, USP8 has the ability to block the anti-A3G activity of Vif molecules from diverse HIV-1 subtypes as well as distant related lentiviral Vif molecules (**Figure 1G** and **Supplementary Figures S2G, H**). Taken together, USP8 targets diverse Vif viral substrate receptors, but not the E3 cellular components or A3G. USP8 antagonized the ability of HIV-1 Vif to suppress the antiviral function of A3G, resulting in an enhanced antiviral activity of A3G against Vif-containing HIV-1. Next, USP8 functions in primary CD4+ T cells and monocyte-derived macrophages (MDMs) from actual patients need to be validated in the future.

Deubiquitinating enzymatic activity of USP8 is required for its antagonism of Vif function. The active site mutant USP8 C786D lost the ability to inhibit Vif-mediated A3G degradation when compared to the WT USP8 (**Figures 2F, G**). During T-cell activation, USP8 is catalytically cleaved by proteolytic enzymes into N-terminal and C-terminal fragments (22). We observed that the C-terminal fragment (C40), but not the N-terminal fragment (N90), maintains deubiquitinase activity (22) and inhibitory activity against HIV-1 Vif (**Figures 2F**, **4G**). In addition, we validated the inhibitory function of USP8 on Vif-mediated A3G polyubiquitination *in vitro* (**Supplementary Figures S3D–F**). Both WT USP8 and functional dominant truncation C40 decreased A3G polyubiquitination, while enzymatic mutation C786D was disabled (**Supplementary Figures S3D–F**).

USP8 is highly expressed in CD4^+^ T cells and myeloid cells, which are the preferred target cells of HIV-1. USP8 is a regulatory factor of the T-cell receptor complex and plays an important role in T-cell function. HIV-1 infection could interfere with T-cell function by disturbing the expression of USP8 (**Figures 4A–F**) or its interaction with Gads5 (**Supplementary Figure S3A**). We discovered that endogenous USP8 expression is significantly reduced in HIV-1-infected H9 and Jurkat cells (**Supplementary Figures S4A, B**). It is interesting to investigate whether any auxiliary proteins of HIV may play a role in it. Unfortunately, we did not observe any significant endogenous USP8 protein expression reduction rescued by Vif-, Vpu-, or Vpx-deficient HIV. Therefore, we speculate that the reduction of USP8 by HIV may not be through its single auxiliary protein. Multiple proteins are potentially involved, including the structural proteins, which needs study in the future.

Interestingly, DUB USP8 exhibits poor activity against HIV-1 Vpr, HIV-1 Vpu, and HIV-2 Rod/SIVmac Vpx. After screening 32 USP functions, we discovered that distinct DUBs inhibit Vpr-mediated HLTF degradation, Vpu-mediated BST-2 degradation (16), and HIV-2 Rod/SIVmacVpx-mediated SAMHD1 degradation (19, 20). Vpx relieves inhibition of HIV-1 infection of myeloid cells mediated by the SAMHD1 protein (19, 20). SAMHD1 restricts reverse transcription of HIV in myeloid cells and resting T cells through its dNTP triphosphohydrolase (dNTPase) activity to repress virus replication. Different from SAMHD1, A3G and BST2/Tetherin all impair HIV-1 progeny virus replication. A3G impairs HIV-1 replication by packaging into virions during virus production and inhibits viral reverse transcription in newly infected target cells (10). BST2/Tetherin inhibits HIV-1 release by directly tethering virions to cells (16, 36). Interestingly, whether Vpr could promote HIV-1 replication in T cells is still disputable, and evidence for its role in cycling T lymphocytes has been sparse. Lahouassa et al. (18) and Yan et al. (37), using a sensitive pairwise replication competition assay, demonstrated that Vpr antagonizes HLTF to promote HIV-1 replication more fitness when coinfected with HIV-1 Vpr defective virus. In our study, the degradation of A3G, BST2, and HLTF was enhanced by HIV/SIV accessory protein when specific USPs (USP8, USP7, and USP33) were knocked down. However, we did not observe any effect on the first-round virus replication, as determined by the intracellular Pr55^Gag^ expression level (**Figures 5E, F**). Importantly, USP7, USP8, or USP33 knocking down all promotes the HIV-1 progeny virus infectivity (**Figures 1D, E** and **Supplementary Figures 5K, L**). Therefore, specific USPs target different HIV/SIV accessory proteins to promote host restriction factors against HIVs/SIVs.

**Figure 6:**
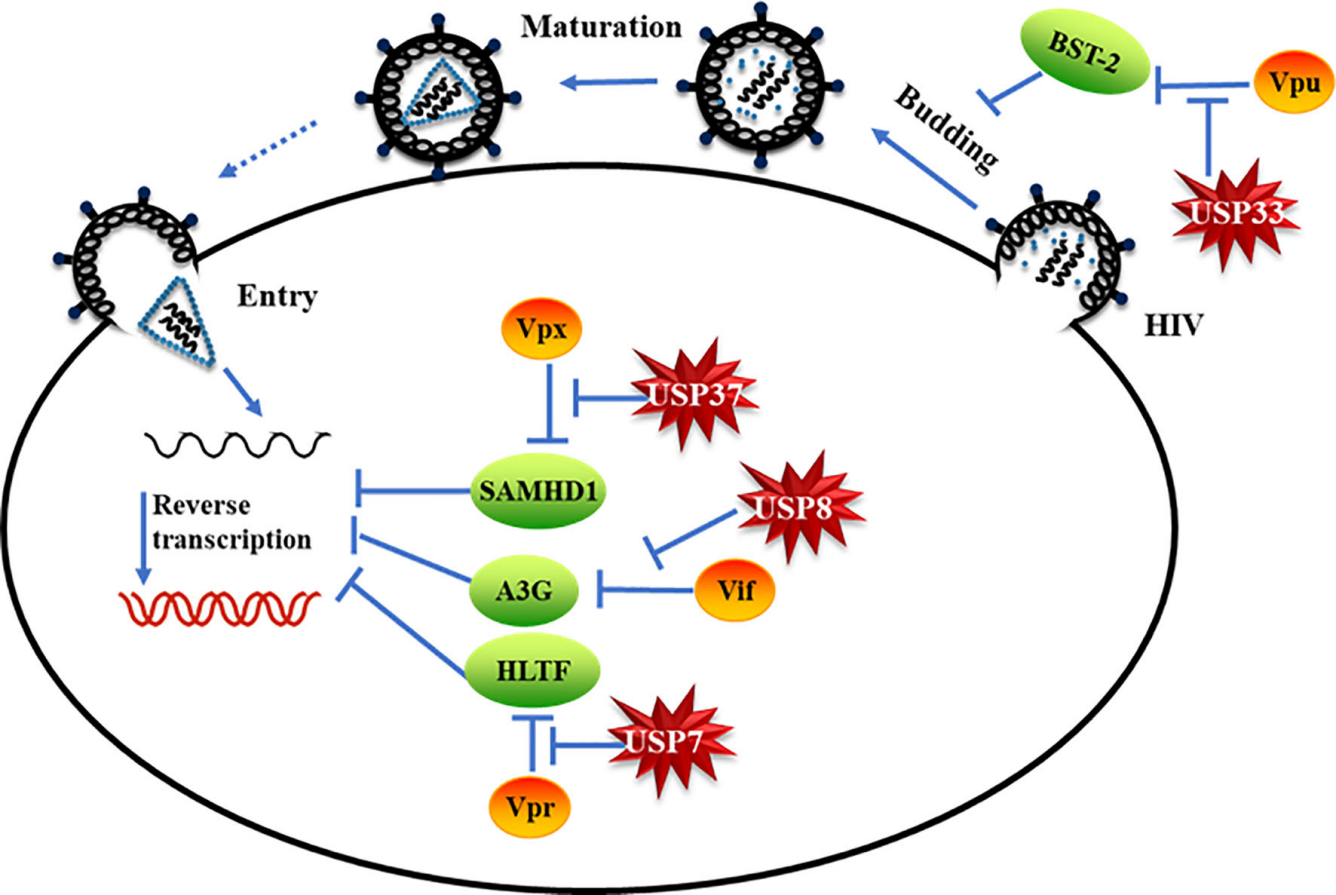
Specific deubiquitinating enzymes suppress viral protein-mediated evasion of cell host restriction. Model of specific deubiquitinating enzymes suppressing HIV/simian immunodeficiency virus (SIV) accessory protein-mediated cell host restriction factor degradation.

Ubiquitin modification of proteins and its reverse process, deubiquitination, regulate all aspects of cellular processes (1, 4, 5). Hijacking host ubiquitin pathways has been linked to the replication of diverse viruses (7). The role of DUBs in the interplay between viruses and the host has not been well characterized. In this study, we demonstrate that DUBs potently inhibit viral evasion of host restriction and viral replication (**Figure 6**). Interestingly, distinct DUBs inhibit different viral proteins with different efficacies. Enhancing the antiviral activity of certain DUBs therefore represents an attractive strategy against HIVs/SIVs.

## Materials and Methods

### Plasmid Construction

USP8, USP8 N90, and USP8 C40 were constructed by PCR amplification from USP8-HA/Flag (#79639; Addgene) and then inserted between the SalI and BamHI sites of a C-terminal HA/Flag tag VR1012 vector. pET28a-USP8 C40 was constructed by PCR amplification from USP8-HA/Flag and then inserted into the pET28a-Plus vector with a 6xHis-tag at the N terminus. pET28a-USP8 C40 C786D was made from pET28a-USP8 C40 by site-directed mutagenesis. All the USP plasmids were purchased from Addgene. The infectious molecular clone pNL4-3 (WT HIV-1) (38), A3G-HA (13), A3G-V5 (13), HIV-1 NL4-3-Vif-HA (Vif-HA) (38), BIV-Vif-HA (39), A3Z2Z3-HA (39), HIV-2RodVpx (40), SIVmac Vpx-HA (40, 41), SAMHD1-HA (41), A3F-V5 (28), A3C-HA (42), A3DE-HA (42), Vpu-Myc (43) and BST-2-HA (43), A3H-hapII-HA (44), and renilla (45) were as previously described. Vifs from different HIV-1 subtypes have been previously described (26). Vpr-HA, HLTF-Flag, Vif (HxB2)-HA, Vif-Myc, and Vpx-Myc were obtained from the Institute of Virology and AIDS Research, First Hospital of Jilin University, and were as previously described (13, 38, 41).

### Cells

HEK293T (CRL-11268; ATCC), HeLa (CRM-CCL-2; ATCC), and TZM-bl (PTA-5659; ATCC) cells were maintained in Dulbecco’s modified Eagle’s medium (DMEM; HyClone) containing 10% heat-inactivated fetal bovine serum (FBS, 04-001-1; Biological Industries) and penicillin/streptomycin. H9 (HTB-176; ATCC), Jurkat (TIB-152; ATCC), and THP1 (TIB-202; ATCC) cells were purchased from the ATCC and maintained in Roswell Park Memorial Institute 1640 (RPMI-1640) medium (HyClone) with 10% FBS and penicillin/streptomycin. The peripheral blood mononuclear cells (PBMCs) were isolated through Ficoll gradient centrifugation, and the CD4^+^ T lymphocytes were then purified from the PBMCs with anti-CD4-specific antibody-coated microbeads (Miltenyi Biotec, Germany) according to the manufacturer’s instructions. CD4^+^ T lymphocytes were maintained in RPMI-1640 medium (HyClone) with 10% FBS and penicillin/streptomycin.

### Transfection

DNA transfection was carried out using Lipofectamine 3000 (Invitrogen) according to the manufacturer’s instructions. H9 and Jurkat cells were transfected using the Amaxa Cell Line Nucleofector Kit V (Lonza, Switzerland) with the program G-014 or X-001 according to the manufacturer’s instructions.

### Reagents and Antibodies

The antibodies used in this study are as follows: β-tubulin monoclonal antibody (NMS-410P; Covance), Anti-CUL5 (sc-13014; Santa Cruz Biotechnology), anti-EloB (sc-1144; Santa Cruz Biotechnology), anti-CBFb (sc-166142; Santa Cruz Biotechnology), anti-Vif (GTX80393; GeneTex), anti-USP8 (A7031; ABclonal), anti-HA (901513; Biolegend), anti-Myc (AHO0052; Invitrogen), anti-V5 (R960-25; Invitrogen), anti-SAMHD1 (TA502024; OriGene), anti-HLTF (14786-1-AP; Proteintech), anti-BST2 (13560-1-AP; Proteintech), anti-A3G (D221663;Sangon Biotech), anti-USP7 (26948-1-AP; Proteintech), anti-USP33 (20445-1-AP; Proteintech), anti-USP37 (18465-1-AP; Proteintech), anti-His (sc-8036; Santa Cruz Biotechnology). CAp24 mAb (1513) was purchased from the NIH AIDS Reagents Program. Secondary antibodies were alkaline phosphatase-conjugated anti-rabbit (115–055–045; Jackson ImmunoResearch) and anti-mouse (115–055–062; Jackson ImmunoResearch), HRP-conjugated anti-rabbit (NA934V; GE) and anti-mouse (sc-2005; Santa Cruz Biotechnology).

### Immunoblot Analysis

For immunoblot analysis of cell-associated proteins, whole cell lysates were prepared as follows: Cells were collected in culture medium and centrifuged at 5,000 rpm for 5 min. Each supernatant was mixed with an appropriate volume of lysis buffer (50 mM Tris–HCl, pH 7.8, with 150 mM NaCl, 1% NP-40, 1% sodium deoxycholate, and 4 mM EDTA) and corresponding 4× loading buffer (8% sodium dodecyl sulfate in 320 mM Tris–HCl, pH 6.8, with 40% glycerol and 0.002% bromophenol blue). Proteins were solubilized by heating for 30 min at 100°C, with occasional vortexing to shear cellular DNA. Cell lysates were subjected to sodium dodecyl sulfate–polyacrylamide gel electrophoresis (SDS–PAGE). Proteins were transferred to NC membranes (10401396; GE Whatman) and reacted with appropriate antibodies as described in the text, e.g., 1:500 rabbit polyclonal anti-USP8 to detect USP8. Membranes were then incubated with the corresponding secondary antibody, and proteins were visualized using a hypersensitive ECL chemiluminescence detection kit (Proteintech) according to the manufacturer’s protocol; the assembled HIV-1 in the culture supernatants was evaluated by immunoblotting in **Figure 1I** and **Supplementary Figure S1F**. HIV-1 in the culture supernatants was filtered through a 0.45-μm filter and mixed with corresponding 4× loading buffer (described above). Proteins were solubilized by heating for 15 min at 100°C with occasional vortexing, and the HIV-1 supernatants were subjected to immunoblotting.

### Co-Immunoprecipitation

In **Figure 3** and **Supplementary Figures S3** and **S4C**, HEK293T cells were co-transfected with an expression vector as indicated. In **Figures 3D, F** and **Supplementary Figure S4C**, cells were treated with 10 mM MG132 (Sigma) 12 h prior to harvesting (12, 46, 47). Cells were harvested and lysed in lysis buffer (50 mM Tris, pH 7.5, with 150 mM NaCl, 1% NP-40, and complete protease inhibitor cocktail tablets) at 4°C for 1 h, then centrifuged at 10,000×g for 30 min. Precleared cell lysates were mixed with anti-V5 antibody (**Figures 3D, F** and **Supplementary Figures S3A**, **S4C**), anti-Myc antibody (**Figures 3A, B**) or anti-HA antibody (**Supplementary Figure S3C**)-conjugated protein G agarose beads and incubated at 4°C overnight. The second day, the beads were washed six times with washing buffer (20 mM Tris, pH 7.5, with 100 mM NaCl, 0.1 mM EDTA, and 0.05% Tween-20) and centrifuged at 800×g for 1 min each time. The beads were eluted with elution buffer (0.1 M glycine-HCl, pH 3.5). The eluted materials were then analyzed by SDS-PAGE and immunoblotting as previously described.

### RT-qPCR

Total RNA was extracted with TRIzol reagent (15596-026; Invitrogen) according to the manufacturer’s instructions. RNA reverse transcription used EasyScript First-Strand cDNA Synthesis SuperMix (AE301; TransGen Biotech) according to the manufacturer’s instructions. The quantitative real-time polymerase chain reaction (qPCR) was carried out on an Mx3005P instrument (Agilent Technologies, Stratagene, USA) using Power SYBR^®^ Green PCR Master Mix (2x) (4367659; ABI). The primers used in this study are as follows: glyceraldehyde 3-phosphate dehydrogenase (*GAPDH*)-F: GCAAATTCCATGGCACCGT; *GAPDH*-R: TCGCCCCACTTGATTTTGG; *USP8*-RT-F: CTGAAAGACTCTCTGAAAGCCT; *USP8*-RT-R: CCTTTCTCTTTGGTCTCACAT. Data were normalized to the housekeeping *GAPDH* gene, and the relative abundance of the transcripts was calculated using Ct models.

### Chemical Synthesis of siRNA

To generate knocking down USP8 cell lines, chemically synthesized short interfering RNA (siRNA) and a nonspecific control were purchased from RiboBio Co. Ltd. (Guangzhou, China). The siUSP8 sequences are as follows: sense, #1: GCATAAAGGTGAAGTGGCA; #2: GAAAACAGGAAGAGAGGAT; #3: GCAAAGAGGGGCAAAGAAA.

### Knockdown Cell Line Construction

USP7/USP8/USP33/USP37-specific shRNAs with the following target sites were cloned in the lenti-retroviral vector pLKO.1-puro (Addgene). The shRNA sequences are as follows: USP7 shRNA: 5-CCGGCCTGGATTTGTGGTTACGTTACTCGAGTAACGTAACCACAAATCCAGGTTTTTG-3; USP8 shRNA: 5-CCGGTAAGAGTTATGTGCACAGTGCCCTCGAGGGCACTGTGCACATAACTCTTTTTTTG-3; USP33 shRNA 5-CCGGTCTCGACAGTGGCTTAATTAACTCGAGTTAATTAAGCCACTGTCGAGATTTTTG-3; USP37 shRNA 5-CCGGCCGGATTTGCAGAAGATGATACTCGAGTATCATCTTCTGCAAATCCGGTTTTTG-3. HEK293T cells were co-transfected with sh-USP7/USP8/USP33/USP37-pLKO.1 or pLKO.1 plus RRE, REV, and VSV-G expression vectors by using Lipofectamine 3000. At 48 h after transfection, supernatants containing packaged lentivirus were harvested and used to infect H9, Hela, or THP1 cells for 96 h as indicated. Puromycin (3 μg/ml for HEK293T, 5 μg/ml for Hela, and 1.5 μg/ml for THP1) was then added into the culture to screen for stable cell lines.

### HIV Infectivity and Detection

HEK293T cells were transfected with pNL4-3 (WT HIV-1) plasmid; 48 h later, the supernatant was collected and filtered through a 0.45-μm filter. Then, cells were infected with pNL4-3 virus in the presence of diethylaminoethyl-dextran hydrochloride (DEAE; 20 μg/ml) for 30 h. The culture medium was changed 30 h after infection and replaced with fresh medium and harvested at the indicated time. HIV-1 infectivity was assessed using TZM-BL indicator cells. LTR-luciferase was activated when TZM-BL cells were infected by HIV-1. TZM-BL cells were seeded in 24-well format plates (2 × 104 cells/well); 24 h later, the cells were infected with the equivalent of 0.5 ng of HIV-1 p24 antigen in the presence of 20 μg/ml DEAE. Cells were collected and lysed after 48 h. LTR-luciferase activity was measured with the Dual-Luciferase Reporter Assay System (E1910; Promega) according to the manufacturer’s protocol.

### *In Vitro* Deubiquitination Assay

Ubiquitinated A3G was isolated from HEK293T cells transfected with expression vectors of Ub-Flag, Vif-HA, and A3G-V5 and then purified from the cell extracts with anti-V5 antibody-conjugated protein G agarose beads. USP8 or its mutant was purified from HEK293T cells overexpressing USP8-HA or its mutant using anti-HA-Agarose antibody in IP buffer [1% (vol/vol) Triton X-100, 50 mM Tris–HCl pH 7.4, 50 mM EDTA, 150 mM NaCl, 10 mM NaF, 10% glycerol, and fresh protease inhibitor cocktail]. For *in vitro* deubiquitination assay, ubiquitinated A3G protein was incubated with USP8 or its mutant in the deubiquitination buffer (20 mM Tris–HCl pH 8.0, 200 mM NaCl, 1 mM EDTA, 10 mM DTT, 5% glycerol) for 1 h at 37°C. The ubiquitinated A3G was analyzed by immunoblotting.

pET28a-USP8 C40 and pET28a-USP8 C40 C786D fusion protein were expressed in the strain of *BL21(DE3)* and purified by metal-affinity chromatography on chelation resin. For *in vitro* deubiquitination assay, ubiquitinated A3G protein was incubated with USP8 C40 or its mutant in the deubiquitination buffer (mentioned above) for the indicated time courses at 37°C. The ubiquitinated A3G was analyzed by immunoblotting.

### Statistical Analysis

Data from the protein quantitative analysis and luciferase reporter assays are presented as means and standard derivations. Differences among groups were analyzed by ANOVA test (Stata Corp., College 251 Station, TX, USA) (NS, not significant; *p < 0.05, **p < 0.01, ***p < 0.001, ****p < 0.0001).

## Data Availability Statement

The original contributions presented in the study are included in the article/**Supplementary Material**. Further inquiries can be directed to the corresponding authors.

## Ethics Statement

Written informed consent was obtained from the individual(s) for the publication of any potentially identifiable images or data included in this article.

## Author Contributions

XY and WG designed the experiments. WG, GL, YR, CZ, and JS performed the experiments. XY, SH, WG, and YR analyzed the data. HL, WWZ, BZ, WYZ, and YY provided technical support. XY, WG, and YR wrote the paper with help from all authors. All authors contributed to the article and approved the submitted version.

## Funding

This work was supported in part by funding from the National Natural Science Foundation of China (81772169; 31970151; 31900457; 31900133, 82172239, 82102384, 81701988; 81772757), the Chinese Ministry of Science and Technology (2018ZX10731-101-001-014), and the National Natural Science Foundation of Zhejiang Province (LQ21C010001).

## Conflict of Interest

The authors declare that the research was conducted in the absence of any commercial or financial relationships that could be construed as a potential conflict of interest.

## Publisher’s Note

All claims expressed in this article are solely those of the authors and do not necessarily represent those of their affiliated organizations, or those of the publisher, the editors and the reviewers. Any product that may be evaluated in this article, or claim that may be made by its manufacturer, is not guaranteed or endorsed by the publisher.

## Supplementary Material

Supplementary Figure 1 Knocking down of USP8 promotes HIV-1 infectivity. **(A)** USP8 was knocked down by lentivirus infection in H9 cells. USP8 expression was analyzed by immunoblotting. **(B) **The effect of USP8 silencing on HIV infectivity in CD4^+^ cell. **(A)** siUSP8 or si control RNA was electro-transfected into CD4^+^cells. After 24 h, H9 cells were infected with WT HIV for another 30 h. The cells were then washed three times with PBS and placed in fresh 1640 medium with 10% FBS. Cell supernatants were then harvested after 48 h of infection. Proteins in the cell lysates were immunoblotted with the corresponding antibodies. Virus infectivity was assessed using TZM-BL indicator cells. **(C, D)** USP8 knockdown promoted Vif-triggered A3G degradation. **(D) **A3G expression from **(C)** was quantified by ImageJ2X. A3G expression alone was normalized to 100%. **(E, F)** WT HIV-1 vector was co-transfected with increasing amounts of USP8 or without USP8 in HEK293T cells. HIV-1 Pr55Gag in cells and supernatants was analyzed by immunoblotting. Tubulin was used as a loading control. **(G)** HIV-1 infectivity was assessed using TZM-bl indicator cells. The process is described in Materials and Methods. **(H)** Screening 32 USPs function on inhibiting Vif-mediated A3G degradation. HEK293T cells were transfected with A3G-V5 and Vif-HA or its empty vector in the presence of 32 USPs. Cells were harvested 48 h after transfection; protein expression in the cell lysates was analyzed by immunoblotting. Quantification of A3G expression from three independent results was analyzed by ImageJ2X. A3G expression alone was normalized to 100%. **(I)** The second-round screening of selective functional USPs in H. Quantification of A3G expression from three independent results was analyzed by ImageJ2X. A3G expression alone was normalized to 100%. Means and standard deviations are presented. Results are representative of n=3 independent experiments. The statistical significance analyses were performed using two-sided unpaired t-tests (NS, not significant, *p < 0.05; **p < 0.01; ***p < 0.001, ****p < 0.0001).

Supplementary Figure 2 USP8 efficiently inhibits Vif-induced degradation of APOBEC3 proteins. **(A, C)** HEK293T cells were co-transfected with expression vector as indicated. Proteins in the cell lysates were immunoblotted with the corresponding antibodies. **(B, D)** Quantification of A3C or A3DE expression was analyzed by ImageJ2X. A3C/A3DE expression alone was normalized to 100%. **(E)** A model of the assembly of the BIV Vif E3 component mediating polyubiquitination and degradation of target proteins. **(F)** Sequence alignment of bovine (NM_001076126.1) and human (BC110590.2) USP8 using DNAMAN 8.0 software. **(G)** USP8 inhibits BIV Vif-induced degradation of A3Z2Z3. HEK293T cells were co-transfected with expression vector as indicated. Proteins in the cell lysates were immunoblotted with the corresponding antibodies. **(H)** Quantification of A3Z2Z3 expression was analyzed by ImageJ2X. Data are representative of at least three independent repeats. The statistical significance analyses were performed using two-tailed unpaired t-tests (***p < 0.001; ****p < 0.0001).

Supplementary Figure 3 Vif affects the association of USP8 and Gads, and USP8 barely interacts with the CRL5 E3-ubiquitin-ligase complex. **(A)** USP8 interacts poorly with Gads in the presence of Vif. USP8-HA was co-transfected with Vif-Myc, Gads-V5 alone, or both, into HEK293T cells. Cell lysates were prepared and immunoprecipitated 48 h after transfection using anti-V5 antibody conjugated to agarose beads. Cell lysates and precipitated samples were analyzed by immunoblotting with the corresponding antibodies. Tubulin was used as the loading control for the cell lysate (n=3). **(B)** Relative binding ability of USP8 and Gads in the presence or absence of Vif. Lane 2 from A was set to 100%. **(C)** USP8-HA or Vif-HA was transfected into HEK293T cells. Cells were treated with 10 mM MG132 12 h prior to harvesting. Cell lysates were prepared and immunoprecipitated overnight using anti-HA agarose beads. Cell lysates and precipitated samples were analyzed by immunoblotting with anti-HA, anti-Cul5, anti-CBFβ, or anti-EloB antibody. Tubulin was used as the loading control for the cell lysate (n=3). **(D)** USP8 deubiquitinates A3G *in vivo*. Ubiquitinated A3G was purified from HEK293T cells transfected with Ub-Flag,Vif-HA and A3G-V5 using anti-V5 affinity purification. HA-tagged USP8, USP8 C40 or USP8^C786D^ was purified from HEK293T cells using anti-HA affinity purification. Ubiquitinated A3G-V5 was incubated with HA-tagged USP8 or USP8^C786D^ for 1h, followed by immunoblotting using antibodies against Ub-Flag and USP8-HA. **(E)** Ubiquitinated A3G was purified from HEK293T cells transfected with Ub-Flag, Vif-HA and A3G-V5 using anti-V5 affinity purification. USP8 C40 recombinant protein was purified by Ni2+-NTA beads. Ubiquitinated A3G-V5 was incubated with His-tagged USP8C40 for indicated times, followed by immunoblotting using antibodies against Ub-Flag and USP8-His. **(F)** Ubiquitinated A3G was purified from HEK293T cells transfected with Ub-Flag, Vif-HA and A3G-V5 using anti-V5 affinity purification. USP8 C40 and USP8 C40^C786D^ recombinant protein was purified by Ni2+-NTA beads. Ubiquitinated A3G-V5 was incubated with His-tagged USP8C40 or USP8 C40^C786D^ for 1h, followed by immunoblotting using antibodies against Ub-Flag and USP8-His. Results from **(A-F)** are representative of n=3 independent experiments.

Supplementary Figure 4 HIV-1 antagonizes USP8 suppression in CD4^+^ cells, and USP8 C40 inhibits HIV-1 Vif-induced A3G polyubiquitination. **(A, B)** HIV-1 suppresses USP8 expression at the protein level. H9 **(A)** and Jurkat **(D)** cells were infected with WT HIV or not for 48 h and 72h. Endogenous USP8 was analyzed by immunoblotting. Virus infection was determined by the presence of Pr55Gag. Tubulin was used as a loading control. USP8 expression was measured by ImageJ2X (mock infection was set to 100%). **(C)** The C terminus of USP8 is important for the inhibition of Vif-induced A3G degradation. HEK293T cells were transfected with Vif-HA, A3G-V5, and Ub-Myc in the presence of WT USP8, a truncation (N909 or C40), or control vector. Cells were treated with 10 mM MG132 for 12 h prior to harvesting. Cell lysates were prepared and immunoprecipitated overnight using anti-V5 antibody conjugated to agarose beads. Cell lysates and precipitated samples were analyzed by immunoblotting with the corresponding antibodies. **(D)** A3G ubiquitination was measured by ImageJ2X; A3G mock was set to 100%. Means and standard deviations are from three independent experiments. The statistical significance analyses were performed using two-tailed unpaired t-tests (NS, not significant; ***p<0.001).

Supplementary Figure 5 **(A)** Model showing how HIV-1 Vpr assembles E3 ubiquitin ligase complexes to target HLTF protein polyubiquitination and degradation. **(B)** Model showing how HIV-1 Vpu assembles E3 ubiquitin ligase complexes to target BST-2 protein polyubiquitination and degradation. **(C)** Model showing how HIV-2/SIV Vpx assembles E3 ubiquitin ligase complexes to target SAMHD1 protein polyubiquitination and degradation. **(D)** Screening 32 USPs function on inhibiting Vpr-mediated HLTF degradation. HEK293T cells were transfected with HLTF-HA and Vpr-HA or its empty vector in the presence of 32 USPs. Cells were harvested 48 h after transfection; protein expression in the cell lysates was analyzed by immunoblotting. Quantification of HLTF expression was analyzed by ImageJ2X. HLTF expression alone was normalized to 100%. **(E)** Screening 32 USPs function on inhibiting Vpu-mediated BST2 degradation. Quantification of BST2 expression was analyzed by ImageJ2X. BST2 expression alone was normalized to 100%. **(F)** Screening 32 USPs function on inhibiting Vpx-mediated SAMHD1 degradation. Quantification of SAMHD1 expression was analyzed by ImageJ2X. SAMHD1 expression alone was normalized to 100%. **(G)** USP8 does not inhibit HIV-1 Vpr-induced degradation of HLTF. HEK293T cells were transfected with expression vector as indicated. Proteins in the cell lysates were immunoblotted with the corresponding antibodies. The relative expression of HLTF was analyzed by ImageJ2X. HLTF expression alone was set to 100%. **(H)** USP8 does not inhibit HIV-1 Vpu-induced BST-2 degradation. **(I, J)** USP8 does not inhibit HIV-2/SIV Vpx-induced SAMHD1 degradation. **(K, L)** Virus infectivity was assessed using TZM-BL indicator cells. shCON virus infectivity was set as 100%. Results from D-J are representative of n=3 independent experiments. Means and standard deviations are from three independent experiments. The statistical significance analyses were performed using two-tailed unpaired t-tests (NS, not significant; **p < 0.01; ***p < 0.001; ****p < 0.0001).
