# Supplementary figures and images for: Specific Deubiquitinating Enzymes Promote Host Restriction Factors Against HIV/SIV Viruses
Source: Front Immunol. 2021 Sep 22;12:740713. doi: 10.3389/fimmu.2021.740713 (PMC8492978; doi:10.3389/fimmu.2021.740713)

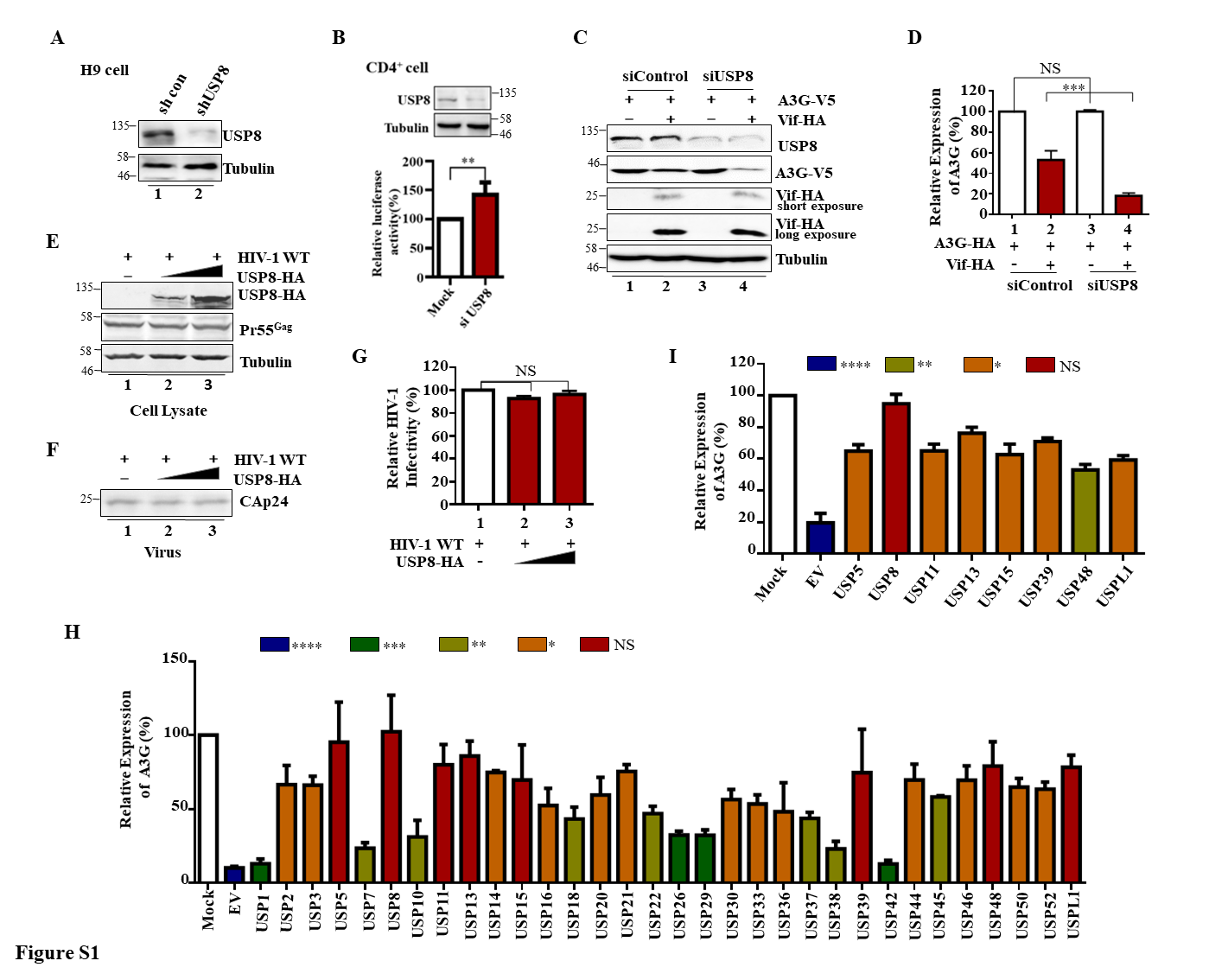

Supplement: Supplementary Figure 1 — Knocking down of USP8 promotes HIV-1 infectivity. (A) USP8 was knocked down by lentivirus infection in H9 cells. USP8 expression was analyzed by immunoblotting. (B) The effect of USP8 silencing on HIV infectivity in CD4+ cell. (A) siUSP8 or si control RNA was electro-transfected into CD4+cells. After 24 h, H9 cells were infected with WT HIV for another 30 h. The cells were then washed three times with PBS and placed in fresh 1640 medium with 10% FBS. Cell supernatants were then harvested after 48 h of infection. Proteins in the cell lysates were immunoblotted with the corresponding antibodies. Virus infectivity was assessed using TZM-BL indicator cells. (C, D) USP8 knockdown promoted Vif-triggered A3G degradation. (D) A3G expression from (C) was quantified by ImageJ2X. A3G expression alone was normalized to 100%. (E, F) WT HIV-1 vector was co-transfected with increasing amounts of USP8 or without USP8 in HEK293T cells. HIV-1 Pr55Gag in cells and supernatants was analyzed by immunoblotting. Tubulin was used as a loading control. (G) HIV-1 infectivity was assessed using TZM-bl indicator cells. The process is described in Materials and Methods. (H) Screening 32 USPs function on inhibiting Vif-mediated A3G degradation. HEK293T cells were transfected with A3G-V5 and Vif-HA or its empty vector in the presence of 32 USPs. Cells were harvested 48 h after transfection; protein expression in the cell lysates was analyzed by immunoblotting. Quantification of A3G expression from three independent results was analyzed by ImageJ2X. A3G expression alone was normalized to 100%. (I) The second-round screening of selective functional USPs in H. Quantification of A3G expression from three independent results was analyzed by ImageJ2X. A3G expression alone was normalized to 100%. Means and standard deviations are presented. Results are representative of n=3 independent experiments. The statistical significance analyses were performed using two-sided unpaired t-tests (NS, not significant [file Image_1.tif]

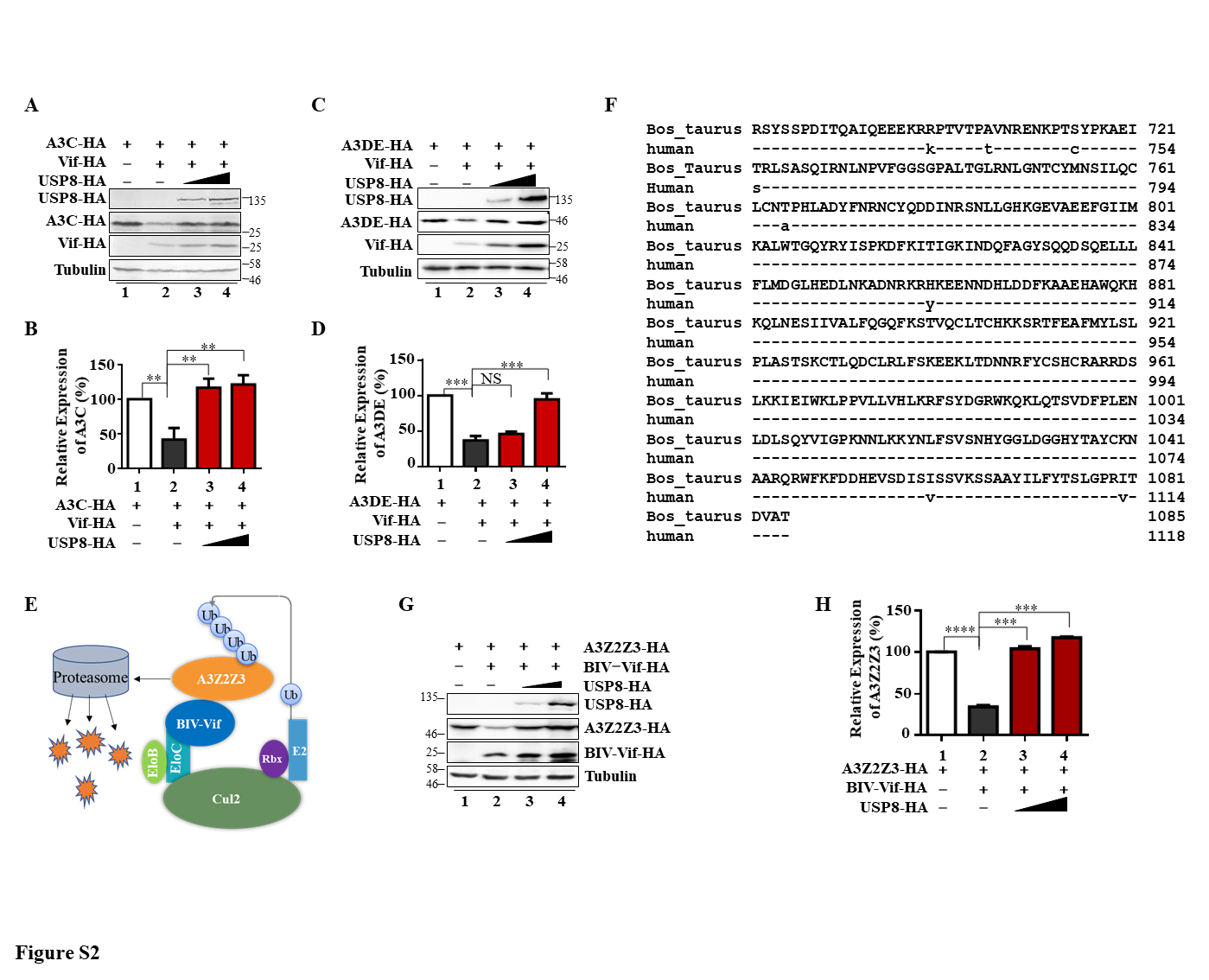

Supplement: Supplementary Figure 2 — USP8 efficiently inhibits Vif-induced degradation of APOBEC3 proteins. (A, C) HEK293T cells were co-transfected with expression vector as indicated. Proteins in the cell lysates were immunoblotted with the corresponding antibodies. (B, D) Quantification of A3C or A3DE expression was analyzed by ImageJ2X. A3C/A3DE expression alone was normalized to 100%. (E) A model of the assembly of the BIV Vif E3 component mediating polyubiquitination and degradation of target proteins. (F) Sequence alignment of bovine (NM_001076126.1) and human (BC110590.2) USP8 using DNAMAN 8.0 software. (G) USP8 inhibits BIV Vif-induced degradation of A3Z2Z3. HEK293T cells were co-transfected with expression vector as indicated. Proteins in the cell lysates were immunoblotted with the corresponding antibodies. (H) Quantification of A3Z2Z3 expression was analyzed by ImageJ2X. Data are representative of at least three independent repeats. The statistical significance analyses were performed using two-tailed unpaired t-tests (***p < 0.001; ****p < 0.0001). [file Image_2.tif]

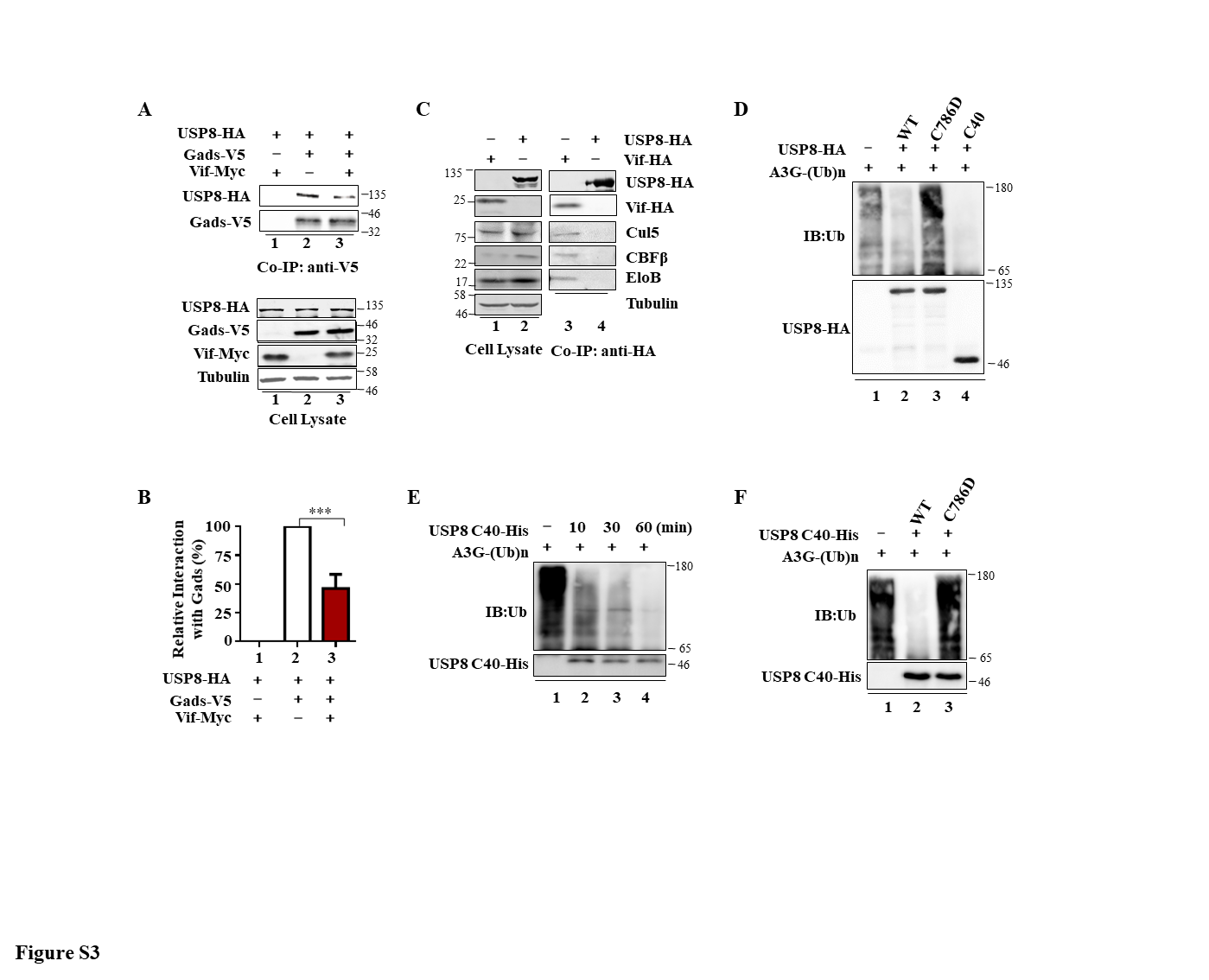

Supplement: Supplementary Figure 3 — Vif affects the association of USP8 and Gads, and USP8 barely interacts with the CRL5 E3-ubiquitin-ligase complex. (A) USP8 interacts poorly with Gads in the presence of Vif. USP8-HA was co-transfected with Vif-Myc, Gads-V5 alone, or both, into HEK293T cells. Cell lysates were prepared and immunoprecipitated 48 h after transfection using anti-V5 antibody conjugated to agarose beads. Cell lysates and precipitated samples were analyzed by immunoblotting with the corresponding antibodies. Tubulin was used as the loading control for the cell lysate (n=3). (B) Relative binding ability of USP8 and Gads in the presence or absence of Vif. Lane 2 from A was set to 100%. (C) USP8-HA or Vif-HA was transfected into HEK293T cells. Cells were treated with 10 mM MG132 12 h prior to harvesting. Cell lysates were prepared and immunoprecipitated overnight using anti-HA agarose beads. Cell lysates and precipitated samples were analyzed by immunoblotting with anti-HA, anti-Cul5, anti-CBFβ, or anti-EloB antibody. Tubulin was used as the loading control for the cell lysate (n=3). (D) USP8 deubiquitinates A3G in vivo. Ubiquitinated A3G was purified from HEK293T cells transfected with Ub-Flag,Vif-HA and A3G-V5 using anti-V5 affinity purification. HA-tagged USP8, USP8 C40 or USP8C786D was purified from HEK293T cells using anti-HA affinity purification. Ubiquitinated A3G-V5 was incubated with HA-tagged USP8 or USP8C786D for 1h, followed by immunoblotting using antibodies against Ub-Flag and USP8-HA. (E) Ubiquitinated A3G was purified from HEK293T cells transfected with Ub-Flag, Vif-HA and A3G-V5 using anti-V5 affinity purification. USP8 C40 recombinant protein was purified by Ni2+-NTA beads. Ubiquitinated A3G-V5 was incubated with His-tagged USP8C40 for indicated times, followed by immunoblotting using antibodies against Ub-Flag and USP8-His. (F) Ubiquitinated A3G was purified from HEK293T cells transfected with Ub-Flag, Vif-HA and A3G-V5 using anti-V5 affinity purification. USP8 C40 and USP [file Image_3.tif]

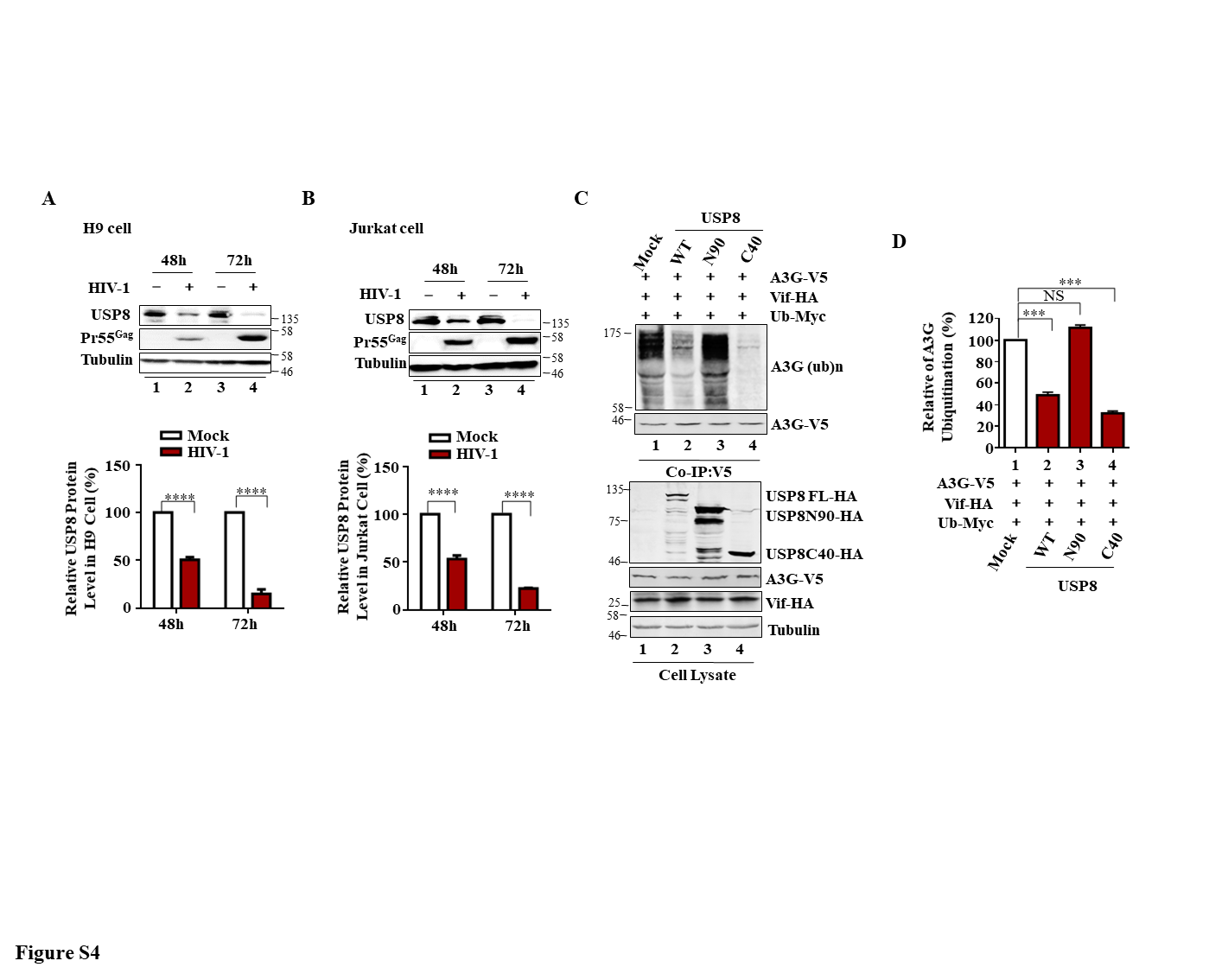

Supplement: Supplementary Figure 4 — HIV-1 antagonizes USP8 suppression in CD4+ cells, and USP8 C40 inhibits HIV-1 Vif-induced A3G polyubiquitination. (A, B) HIV-1 suppresses USP8 expression at the protein level. H9 (A) and Jurkat (D) cells were infected with WT HIV or not for 48 h and 72h. Endogenous USP8 was analyzed by immunoblotting. Virus infection was determined by the presence of Pr55Gag. Tubulin was used as a loading control. USP8 expression was measured by ImageJ2X (mock infection was set to 100%). (C) The C terminus of USP8 is important for the inhibition of Vif-induced A3G degradation. HEK293T cells were transfected with Vif-HA, A3G-V5, and Ub-Myc in the presence of WT USP8, a truncation (N909 or C40), or control vector. Cells were treated with 10 mM MG132 for 12 h prior to harvesting. Cell lysates were prepared and immunoprecipitated overnight using anti-V5 antibody conjugated to agarose beads. Cell lysates and precipitated samples were analyzed by immunoblotting with the corresponding antibodies. (D) A3G ubiquitination was measured by ImageJ2X; A3G mock was set to 100%. Means and standard deviations are from three independent experiments. The statistical significance analyses were performed using two-tailed unpaired t-tests (NS, not significant; ***p<0.001). [file Image_4.tif]

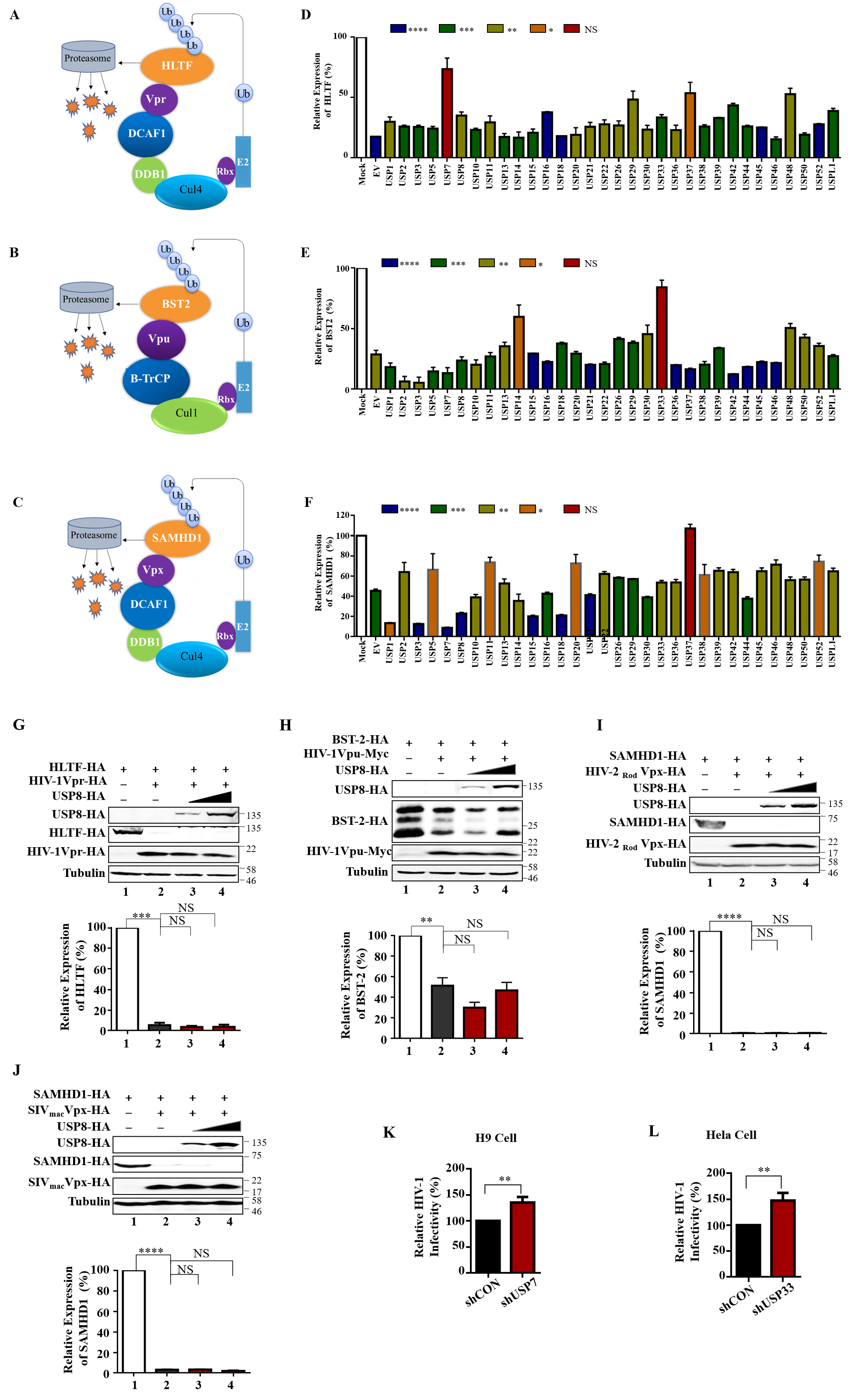

Supplement: Supplementary Figure 5 — (A) Model showing how HIV-1 Vpr assembles E3 ubiquitin ligase complexes to target HLTF protein polyubiquitination and degradation. (B) Model showing how HIV-1 Vpu assembles E3 ubiquitin ligase complexes to target BST-2 protein polyubiquitination and degradation. (C) Model showing how HIV-2/SIV Vpx assembles E3 ubiquitin ligase complexes to target SAMHD1 protein polyubiquitination and degradation. (D) Screening 32 USPs function on inhibiting Vpr-mediated HLTF degradation. HEK293T cells were transfected with HLTF-HA and Vpr-HA or its empty vector in the presence of 32 USPs. Cells were harvested 48 h after transfection; protein expression in the cell lysates was analyzed by immunoblotting. Quantification of HLTF expression was analyzed by ImageJ2X. HLTF expression alone was normalized to 100%. (E) Screening 32 USPs function on inhibiting Vpu-mediated BST2 degradation. Quantification of BST2 expression was analyzed by ImageJ2X. BST2 expression alone was normalized to 100%. (F) Screening 32 USPs function on inhibiting Vpx-mediated SAMHD1 degradation. Quantification of SAMHD1 expression was analyzed by ImageJ2X. SAMHD1 expression alone was normalized to 100%. (G) USP8 does not inhibit HIV-1 Vpr-induced degradation of HLTF. HEK293T cells were transfected with expression vector as indicated. Proteins in the cell lysates were immunoblotted with the corresponding antibodies. The relative expression of HLTF was analyzed by ImageJ2X. HLTF expression alone was set to 100%. (H) USP8 does not inhibit HIV-1 Vpu-induced BST-2 degradation. (I, J) USP8 does not inhibit HIV-2/SIV Vpx-induced SAMHD1 degradation. (K, L) Virus infectivity was assessed using TZM-BL indicator cells. shCON virus infectivity was set as 100%. Results from D-J are representative of n=3 independent experiments. Means and standard deviations are from three independent experiments. The statistical significance analyses were performed using two-tailed unpaired t-tests (NS, not significant; **p < 0.01; ***p < 0.001; [file Image_5.tif]
